# Supplementary material for: Surprising diversity of new plasmids in bacteria isolated from hemorrhoid patients
Source: PeerJ. 2024 Aug 30;12:e18023. doi: 10.7717/peerj.18023 (PMC11368089; doi:10.7717/peerj.18023)
Supplement: Supplemental Information 1 [file peerj-12-18023-s001.pdf]

STROBE Statement—checklist of items that should be included in reports of observational studies

	Item No.	Recommendation	Page No.	Relevant text from manuscript
**Title and abstract**	1	(*a*) Indicate the study's design with a commonly used term in the title or the abstract	1	Surprising diversity of new plasmids in bacteria isolated from hemorrhoid patients
		(*b*) Provide in the abstract an informative and balanced summary of what was done and what was found	1-2	Forty-nine bacterial strains were isolated from seven hemorrhoid patients. Third generation Nanopore sequencing was performed to obtain high quality whole genome sequences. Out of the 31 plasmids found in the strains, 15 new plasmids were discovered
**Introduction**				
Background/rationale	2	Explain the scientific background and rationale for the investigation being reported	2	Recent investigations showed differed signatures of bacterial community structures in hemorrhoids, suggesting possible influence of bacteria in the development of hemorrhoid
Objectives	3	State specific objectives, including any prespecified hypotheses	2	This work aims at addressing the microbes in hemorrhoid-suffering patients issue by whole genome sequencing analysis of bacterial strains isolated form seven hemorrhoid patients
**Methods**				

Study design	4	Present key elements of study design early in the paper	3	Bacteria were taken from seven different sites on the patient,and using third-generation sequencing technology
Setting	5	Describe the setting, locations, and relevant dates, including periods of recruitment, exposure, follow-up, and data collection	3-4	Seven hemorrhoid patients who underwent surgery at Qilu Hospital (Qingdao) were recruited from March to July 2023
Participants	6	(*a*) *Cohort study*—Give the eligibility criteria, and the sources and methods of selection of participants. Describe methods of follow-up<br>*Case-control study*—Give the eligibility criteria, and the sources and methods of case ascertainment and control selection. Give the rationale for the choice of cases and controls<br>*Cross-sectional study*—Give the eligibility criteria, and the sources and methods of selection of participants	3	A total of seven hemorrhoid patients were recruited for the isolation of bacterial strains. Seven different sites on the patient were swabbed with a sterile cotton swab
		(*b*) *Cohort study*—For matched studies, give matching criteria and number of exposed and unexposed<br>*Case-control study*—For matched studies, give matching criteria and the number of controls per case	3	Samples were taken from seven hemorrhoid patients who were subject to surgery in Qilu Hospital (Qingdao). Samples from seven sites were taken from each patient for culture.
Variables	7	Clearly define all outcomes, exposures, predictors, potential confounders, and effect modifiers. Give diagnostic criteria, if applicable	/	/
Data sources/ measurement	8*	For each variable of interest, give sources of data and details of methods of assessment (measurement). Describe comparability of assessment methods if there is more than one group	/	/
Bias	9	Describe any efforts to address potential sources of bias	3	Seven different sites (healthy buttock skin, anal skin, the outer side of the hemorrhoid, the inner side of the hemorrhoid, healthy anal gland, feces, and the hemorrhoid tissue)

Study size	10	Explain how the study size was arrived at	/	/

Continued on next page

Quantitative variables	11	Explain how quantitative variables were handled in the analyses. If applicable, describe which groupings were chosen and why	3	Seven different sites (healthy buttock skin, anal skin, the outer side of the hemorrhoid, the inner side of the hemorrhoid, healthy anal gland, feces, and the hemorrhoid tissue) on the patient were swabbed with a sterile cotton swab and then immersed in the preservation solution
Statistical methods	12	(*a*) Describe all statistical methods, including those used to control for confounding	/	/
		(*b*) Describe any methods used to examine subgroups and interactions	/	/
		(*c*) Explain how missing data were addressed	/	/
		(*d*) *Cohort study*—If applicable, explain how loss to follow-up was addressed<br>*Case-control study*—If applicable, explain how matching of cases and controls was addressed<br>*Cross-sectional study*—If applicable, describe analytical methods taking account of sampling strategy	/	/
		(*e*) Describe any sensitivity analyses	/	/
**Results**				
Participants	13*	(a) Report numbers of individuals at each stage of study—eg numbers potentially eligible, examined for eligibility, confirmed eligible, included in the study, completing follow-up, and analysed	3-4	A total of seven hemorrhoid patients were recruited. 49 strains were obtained
		(b) Give reasons for non-participation at each stage	/	/
		(c) Consider use of a flow diagram	/	/
Descriptive data	14*	(a) Give characteristics of study participants (eg demographic, clinical, social) and information on exposures and potential confounders	/	Provided in Table S1: average age 41, male: female:4:3
		(b) Indicate number of participants with missing data for each variable of interest	/	/
		(c) *Cohort study*—Summarise follow-up time (eg, average and total amount)	/	/
Outcome data	15*	*Cohort study*—Report numbers of outcome events or summary measures over time	/	/
		Case-control study—Report numbers in each exposure category, or summary measures of exposure	/	/
		Cross-sectional study—Report numbers of outcome events or summary measures	/	/

Main results	16	(*a*) Give unadjusted estimates and, if applicable, confounder-adjusted estimates and their precision (eg, 95% confidence interval). Make clear which confounders were adjusted for and why they were included	/	/
		(*b*) Report category boundaries when continuous variables were categorized	/	/
		(*c*) If relevant, consider translating estimates of relative risk into absolute risk for a meaningful time period	/	/

Continued on next page

Other analyses	17	Report other analyses done—eg analyses of subgroups and interactions, and sensitivity analyses	/	/
**Discussion**				
Key results	18	Summarise key results with reference to study objectives	8	With high quality whole genome sequences, we were able to identify 15 new plasmids.
Limitations	19	Discuss limitations of the study, taking into account sources of potential bias or imprecision. Discuss both direction and magnitude of any potential bias	/	/
Interpretation	20	Give a cautious overall interpretation of results considering objectives, limitations, multiplicity of analyses, results from similar studies, and other relevant evidence	8	Surprising diversity of new plasmids in bacteria isolated from hemorrhoid patients
Generalisability	21	Discuss the generalisability (external validity) of the study results	8	This work shows that we have been underestimating plasmid diversities in human-carrying microbes, and as whole genome sequencing has been cheaper and cheaper, surveillance of plasmids is worthy attention in the future.
**Other information**				
Funding	22	Give the source of funding and the role of the funders for the present study and, if applicable, for the original study on which the present article is based	/	Provided in the system: This work was supported by the Foundation of Qingdao Key Health Discipline Development Fund under grand number ODZDZK-2022098, the National Key Research and Development Program of China under grant number 2022YFE0199800; Key R&D Program of Shandong Province under grant number 2020CXGC011305; and the National Natural Science

			Foundation of China under grant number 82271658

*Give information separately for cases and controls in case-control studies and, if applicable, for exposed and unexposed groups in cohort and cross-sectional studies.

Note: An Explanation and Elaboration article discusses each checklist item and gives methodological background and published examples of transparent reporting. The STROBE checklist is best used in conjunction with this article (freely available on the Web sites of PLoS Medicine at http://www.plosmedicine.org/, Annals of Internal Medicine at http://www.annals.org/, and Epidemiology at http://www.epidem.com/). Information on the STROBE Initiative is available at www.strobe-statement.org.
